# Comparison of computational chemistry methods for the discovery of quinone-based electroactive compounds for energy storage

**DOI:** 10.1038/s41598-020-79153-w

**Published:** 2020-12-17

**Authors:** Qi Zhang, Abhishek Khetan, Süleyman Er

**Affiliations:** 1 DIFFER—Dutch Institute for Fundamental Energy Research, De Zaale 20, 5612 AJ Eindhoven, The Netherlands; 2 CCER—Center for Computational Energy Research, De Zaale 20, 5612 AJ Eindhoven, The Netherlands; 3 Department of Applied Physics, Eindhoven University of Technology, 5600 MB Eindhoven, The Netherlands

**Keywords:** Energy, Computational chemistry, Density functional theory

## Abstract

High-throughput computational screening (HTCS) is a powerful approach for the rational and time-efficient design of electroactive compounds. The effectiveness of HTCS is dependent on accuracy and speed at which the performance descriptors can be estimated for possibly millions of candidate compounds. Here, a systematic evaluation of computational methods, including force field (FF), semi-empirical quantum mechanics (SEQM), density functional based tight binding (DFTB), and density functional theory (DFT), is performed on the basis of their accuracy in predicting the redox potentials of redox-active organic compounds. Geometry optimizations at low-level theories followed by single point energy (SPE) DFT calculations that include an implicit solvation model are found to offer equipollent accuracy as the high-level DFT methods, albeit at significantly lower computational costs. Effects of implicit solvation on molecular geometries and SPEs, and their overall effects on the prediction accuracy of redox potentials are analyzed in view of computational cost versus prediction accuracy, which outlines the best choice of methods corresponding to a desired level of accuracy. The modular computational approach is applicable for accelerating the virtual studies on functional quinones and the respective discovery of candidate compounds for energy storage.

## Introduction

Commercial utilization of intermittent renewable energy sources, such as solar and wind, requires large-scale, low-cost, and durable energy storage technologies to balance the mismatch between energy supply and demand. Redox flow batteries (RFBs) are recognized as prime candidates for large-scale and variable-term storage of electrical energy^1–3^. RFBs have external storage tanks that store the liquid-phase redox-active electrolyte materials separated from the electrochemical reaction cells. This unique design feature is advantageous as it decouples the battery’s power and energy density scaling, while also facilitating easier maintenance and recycling^2^.

Conventional RFBs are operated using metal-based electrolyte materials, such as vanadium, iron, zinc, lead and chromium^4^. They, however, face technical challenges of ion crossover through the membranes and sluggish reaction kinetics^5^. Additionally, the cost of active materials and the risks associated with metal toxicity have been hindering widespread deployment of metal-based RFBs^6,7^. RFBs employing organic redox-active materials offer a promising alternative to metal-based electrolytes, as they can be sourced from earth-abundant elements and modified further structurally to tune their key battery-relevant properties^1,2^. The emerging classes of organic redox-active RFB compounds consist of quinones^3^, viologens^8,9^, alloxazines^10,11^, phenazines^12,13^, and nitroxide radicals^14^. Quinones are ubiquitous in nature^15^, and with their fast redox kinetics^16,17^ and tunable properties owing to their chemical diversity^1,18^, they are increasingly being utilized as electroactive materials in advanced RFB technologies. In recent years, an increasing effort has been made to develop aqueous RFBs (ARFBs) that use quinones as electroactive materials, including the functionalized forms of benzoquinones^19,20^, naphthoquinones^17,19^, and anthraquinones^19,21^. Research has shown that these molecules undergo a coupled two-electron two-proton redox reaction in aqueous media^22^. However, these molecules offer low energy densities in practical ARFBs as they are not very soluble and their half-cell redox potentials are not close to 0 V versus SHE, which is desired for ARFB anolytes^19,23,24^. Therefore, a major challenge for organic ARFBs is to tune the properties of the electroactive compounds to meet the practical requirements of high power and high energy density batteries. To develop an ARFB with a large cell voltage, maximizing the redox potential window of quinone-based compounds is essential. Recent experimental and computational studies show that the redox potential of organic ARFBs are significantly influenced by functionalizing them with electron-withdrawing/donating groups^1,2^. Assary et al.^25^ and Aspuru-Guzik et al.^26,27^ used HTCS methods for creating virtual libraries of candidate electroactive compounds populated with the functionalized compounds of quinones and predicting their redox properties. These studies utilized robust quantum chemical calculations to estimate the thermodynamic properties of compounds and identify the most promising candidates. Thus, they demonstrated the usefulness of hierarchical HTCS methods in accelerating the property predictions of redox-active molecules. Using quantum chemical calculations to predict the redox properties is, however, a practically challenging task. The approach is particularly not well-suited for HTCS studies on a huge space of conceivable molecules. Therefore, there is a need to determine the trade-offs between the prediction accuracy and the computational cost. While there has been a significant increase in the number of HTCS efforts for RFBs^25–32^, to the best of our knowledge, an analysis of the factors that affect prediction accuracy, such as the level of theory for optimization of molecular geometry, inclusion/exclusion of solvation effects, and the level of theory for the calculation of chemical descriptors, are not available in the current literature.

Here, we systematically evaluate the performance of different computational methods, including DFT, DFTB^33^, and SEQM^34^. We compare them based on their accuracy in predicting the experimentally measured redox potentials of quinones obtained from four different sources. In addition, we make first-order comparisons of the computational cost of these approaches to offer the best approach for the prediction of redox potentials. Our results provide new insights on the factors that influence the prediction performance of computational methods. The findings are expected to be useful for both customary and HTCS efforts that are aimed at studying of redox-active molecules for RFBs, and also beyond ARFB compounds that take part in bio- and electro-chemical conversion reactions.

### Computational workflow

**Figure 1:**
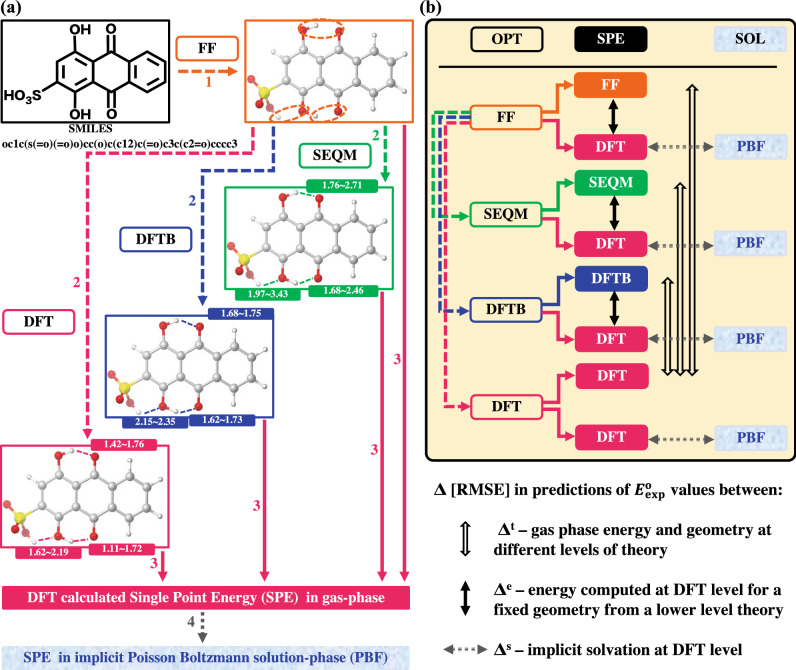
(**a**) Schematic showing the chain of operations for geometry optimization (OPT) and single point energy (SPE) calculations at different levels of theory (**b**) A graphical summary of the various levels of approximations used to estimate $\Delta{E}_{\mathrm{rxn}}^{\mathrm{DFT}}$. The hollow black arrows with symbol Δ^t^ represent the difference between gas-phase reaction energies of the optimized geometries obtained at different levels of theory. The solid black arrows with symbol Δ^e^ represent the difference between energies computed using DFT on a fixed geometry obtained from a lower-level theory and energies computed at that given level of theory. The dotted gray arrows represent the solvation effect from gas-phase SPE to solution-phase SPE, when the implicit solvation model is considered. For both (**a**,**b**), the text boxes with no background color represent geometry optimizations; the boxes with colored background represent SPE calculations; the boxes with water bubbles in the background represent solution-phase SPE calculations using the implicit aqueous solvent model.

We developed a systematic workflow to make generalizable and consistent comparisons between the different computational approaches. As shown in Fig. 1, the starting point in the workflow for any molecule is its SMILES representation^35^, which is a widely used form of graph representation and can easily be generated for any given molecule. The SMILES representation is at first converted to a two-dimensional (2D) geometrical representation using a SMILES interpreter. Next,

1. The 2D representation is converted to a three-dimensional (3D) geometry by applying the geometry optimization (OPT) scheme of OPLS3e FF^36^ to identify the lowest energy 3D conformer. As shown in Fig. 1b, the FF level geometry is the starting point for all approaches.
2. The 3D geometry is further optimized in the gas-phase at three different levels of theory, namely: SEQM, DFTB, and DFT. For SEQM and DFT, geometry optimizations have also been performed in an implicit aqueous-phase, but for simplicity they are not shown in Fig. 1. This step yields different 3D geometries and the corresponding SPE of the compounds.
3. Next, SPEs of all the different 3D geometries are calculated using different DFT functionals. This step yields energy values that are directly comparable but are obtained from geometry optimizations that have been performed at four different levels of theory.
4. Lastly, for the geometries obtained from the optimizations in the gas-phase, the SPEs are recalculated, this time by including the effect of the aqueous medium (SOL) implicitly by using the Poisson–Boltzmann solvation model (PBF)^37,38^.

## Results and discussions

### Comparison of DFT methods

Since DFT is the highest level of theory considered in the current study, we begin with a discussion of the performance of the various DFT functionals, also with an aim to use them as performance benchmarks for low-level methods. Initially, we briefly discuss the performance of $\Delta{U}_{\mathrm{rxn}}$ and $\Delta{G}_{\mathrm{rxn}}^{\mathrm{o}}$ as chemical descriptors to predict redox potentials. For this purpose, DFT energy calculations using the PBE functional are performed, first for optimizing geometries in gas-phase and then for calculating single point energies in an implicit aqueous-phase. The calibration performances of $\Delta{U}_{\mathrm{rxn}}$ (RMSE = 0.049 V, R^2^ = 0.978) and $\Delta{G}_{\mathrm{rxn}}^{\mathrm{o}}$ (RMSE = 0.048 V, R^2^ = 0.979) are very similar, as shown in Supplementary Fig. S1. Inclusion of ZPE in $\Delta{U}_{\mathrm{rxn}}$, as well as entropic effects in $\Delta{G}_{\mathrm{rxn}}^{\mathrm{o}}$, is only marginally better than using $\Delta{E}_{\mathrm{rxn}}$ (RMSE = 0.051 V, R^2^ = 0.977). Therefore, we consider that the effects of including these terms are not significant enough from HTCS perspective. Accordingly, all the following discussions in this work consider $\Delta{E}_{\mathrm{rxn}}$ as the descriptor.

**Figure 2:**
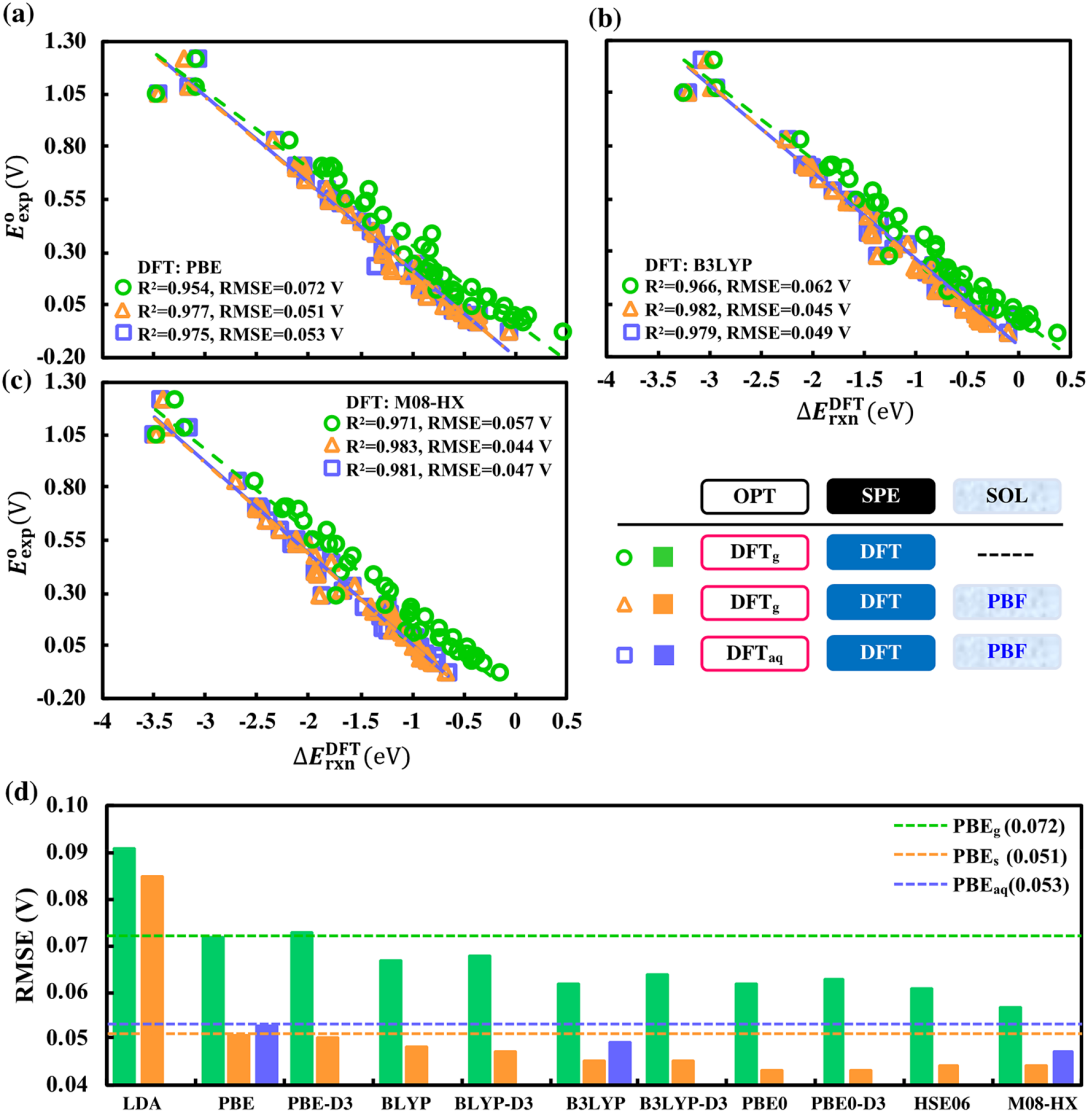
Performance comparisons of different exchange–correlation functionals in predicting the experimentally measured redox potentials, ${E}_{\mathrm{exp}}^{\mathrm{o}}$. The scatter plots (**a**–**c**) show linear correlations of the DFT calculated energy difference, $\Delta{E}_{\mathrm{rxn}}^{\mathrm{DFT}}$, versus ${E}_{\mathrm{exp}}^{\mathrm{o}}$, for the three representative functionals of (**a**) PBE, (**b**) B3LYP, and (**c**) M08-HX. The bar plot (**d**) shows the RMSE for all the functionals considered. The color green represents both OPT and SPE in gas-phase, the color orange represents OPT in gas-phase followed by SPE with SOL, and the color blue represents both OPT and SPE with SOL, as also summarized in a tabular format. In (**d**), the horizontal dashed green line represents PBE_g_ (RMSE = 0.072 V, R^2^ = 0.954) benchmark, the horizontal dashed blue line represents PBE_aq_ (RMSE = 0.053 V, R^2^ = 0.975) benchmark, and the horizontal dashed orange line represents PBE_s_ (RMSE = 0.051 V, R^2^ = 0.977) benchmark.

First, we discuss linear calibrations of three representative DFT functionals of PBE (Fig. 2a), B3LYP (Fig. 2b), and M08-HX (Fig. 2c). The results shown in Fig. 2 have been obtained by using three kinds of DFT calculated reaction energies $\Delta{E}_{\mathrm{rxn}}=\Delta{E}_{\mathrm{rxn}}^{\mathrm{DFT}}$, against the experimentally measured redox potentials (${E}_{\mathrm{exp}}^{\mathrm{o}}$) as follows: (1) OPT in gas-phase without calculation of SPE in SOL, (2) OPT in gas-phase and a following SPE in SOL, and (3) both OPT and SPE in SOL.

On comparing RMSE and R^2^ data, the following observations are made:

1. When using $\Delta{E}_{\mathrm{rxn}}^{\mathrm{DFT}}$ from only gas-phase optimized geometry and SPE, PBE is the least accurate functional at the GGA level with RMSE = 0.072 V, R^2^ = 0.954. Nevertheless, the results show that at any DFT level, it is possible to predict ${E}_{\mathrm{exp}}^{\mathrm{o}}$ for quinone-based molecules within a range of common experimental errors (~ 0.1 V).
2. Upon the inclusion of solvation effects using an implicit model on the gas-phase geometries, all the three functionals show a decrease in their RMSE values. The percentage decrease in error is highest for PBE (30%) and lowest for M08-HX (23%).
3. Remarkably for all the three considered functionals, full geometry optimizations and energy calculations in an implicit solvation model yield slightly worse results than their counterparts in which geometries are optimized in gas-phase. The RMSEs increase by 0.002–0.004 V, indicating that there is no real added value of performing geometry optimizations with implicit solvation, not to mention that they are also computationally more demanding.

Based on the findings above, we evaluate the performances of eight other DFT functionals without considering geometry optimizations in an implicit solution-phase. In Fig. 2d and Supplementary Fig. S2, a summary of the performance of all the DFT functionals considered in this work is presented using bar plots for RMSE and R^2^, respectively. When compared under the same set of conditions, all functionals, with the exception of LDA, show a similar performance. The PBE0/PBE0-D3, HSE06 and M08-HX functionals show a highly similar performance when using implicit solvation on gas-phase optimized geometries, which are followed by the other hybrid functionals of B3LYP/B3LYP-D3, and then by the PBE/PBE-D3 and BLYP/BLYP-D3 GGA functionals. The addition of D3-dispersion corrections makes hardly any difference on either of the hybrid or the GGA functional calculated results. For all further comparisons in this work, we choose, among the compared exchange–correlation functionals, PBE as the benchmark DFT functional as it offers the best compromise between prediction accuracy and computational cost. We note that, the different DFT functionals have been compared here purely on the basis of their performance in predicting the measured potentials. We also note that, functionals constructed with higher degrees of empiricism, such as the Minnesota density functionals^39^, are aimed at producing better values for a chosen set of physically observable properties. In this regard, it is not surprising that the M08-HX performs best among the functionals, as it is heavily parametrized to show good performance for thermochemistry. However, it must also be kept in mind that such heavily parametrized functionals tend to produce less accurate electron densities than the ones with little to no empiricism in their design (e.g. the PBE functional)^40^.

## Discussion

As shown in Fig. 2d, the DFT LDA data is not as good as the GGA and the hybrid method calculated data. Additionally, there is a positive impact on the prediction accuracies due to the inclusion of implicit solvation in calculations of $\Delta{E}_{\mathrm{rxn}}^{\mathrm{DFT}}$. The effect can be attributed to a better accounting of the −OH groups’ interactions with the surrounding aqueous environment in the hydroquinone products^32^. Surprisingly, optimizing geometries with implicit solvation slightly worsens the prediction accuracies. This can be attributed to multiple factors. First, it is possible that the PBF solvation model is not accurate enough to improve the gas-phase geometry. Secondly, there might be a serendipitous cancellation of errors when using the gas-phase geometry that is affected by the changes in the geometry due to the implicit solvation model in use. Additionally, $\Delta{E}_{\mathrm{rxn}}$ is used as an approximation for $\Delta{G}_{\mathrm{rxn}}^{\mathrm{o}}$, and accounting for the ignored pressure–volume and entropy terms from Eq. (6) might result in better prediction accuracies when optimizing the geometries in solution. In the work of Kim et al.^32^ it was shown that the reduction potentials of Anthraquinones in acidic aqueous solutions are strongly influenced by specific interactions with molecules in solvent environment. In aqueous solution, they found that using DFT (ωB97X-D/6-31G^*^) with implicit solvation (PCM(Bondi)) for geometry optimizations yields good results, except for the redox couples that have strong intramolecular hydrogen bond interactions. They evaluated a total of 19 Anthraquinones and reported a mean absolute deviation (MAD) of 0.194 V for three outliers that showed strong intramolecular hydrogen bond interactions. This value was more than five times the MAD value of the remaining 16 redox couples (0.037 V). Further, they showed that QM/MM calculations (with the TIP3P force field for explicit water molecules) alleviate the overestimation and lead to a more balanced treatment of solute–solvent interactions. Accordingly, using a QM/MM model, the correlation between theory and experiment data had a MAD of 0.033 V. In the current work, we performed a similar analysis on our calibration data set of 43 molecule pairs. We found that there are only 12 molecules, with IDs: 1, 2, 3, 4, 5, 6, 8, 9, 16, 35, 37 and 39 shown in Supplementary Table S1, without any possibility of strong intramolecular hydrogen bond interactions due to the neighboring positions found in the hydroquinone versions of the molecules. Surprisingly, we found that when using implicit aqueous solvation during geometry optimizations, the MADs for the predicted redox potentials were 0.039 V for the 12 molecules and 0.037 V for the remaining 31 molecules. We note that these MADs are very similar and the difference between the two groups is only in the third decimal digit. Therefore, we cannot confirm that the explanation provided by Kim et al. also applies to the methods used in this work. At the same time, it must be noted that Kim et al. used only Anthraquinones (3-ring molecules) for their analysis, whereas this work considers a wide variety of quinone molecules (from 1 to 3 rings), including those with the C=O groups at the 1,2 positions on the compounds. Further, Kim et al. employed the PCM (Bondi) implicit solvation model, which is different from the PBF model used in the current work. These differences, as well as the difference in the calibration data, make it hard to ascertain the exact origin of the disparities between this work and the work of Kim et al.

Another important aspect of the calibration of molecules that needs to be considered is the effect of ionization of sulfonic acid groups, as they are prone to dissociation in aqueous media. In the calibration set of 43 molecule pairs used in the current work, there are 18 molecules that contain –SO_3_H groups, with IDs: 7, 8, 9, 10, 11, 12, 13, 30, 31, 32, 33, 34, 35, 36, 37, 41, 42 and 43 as shown in Supplementary Table S1. In the framework of the best performing scheme, i.e. PBE_s_, the calculated MAD for these 18 molecules is 0.047 V, which is approximately 50% higher than the MAD of the remaining 25 molecules (0.032 V). Clearly, the ionization of sulfonic groups has adverse effects on the prediction accuracies. Although the effect is not significant from a perspective of HTCS studies, we recommend the inclusion of explicit water molecules, such as in a QM/MM formalism, when extremely accurate values for the sulfonic group decorated quinone redox potentials are sought^32^.

In order to understand the existence of functional group dependent rational trends, we selected anthraquinone (Molecule ID 5 in Supplementary Table S1) as the base molecule from our calibration set and found six derivatives with –SO_3_H (Supplementary Fig. S3a) and six derivatives with –OH functional groups (Supplementary Fig. S3b). Molecules with mixed functional groups were excluded. As shown in Supplementary Fig. S3a, an increase in the total number of –SO_3_H groups leads to an increase in the redox potentials; whereas an increase in the total number of –OH groups leads to a decrease in the redox potentials, as shown in Supplementary Fig. S3b. These trends are very much in line with the previous knowledge that the electron-withdrawing groups are known to increase the redox potential and vice-versa^26^. It must be noted that the correlation for –SO_3_H is weak, indicating that not only the type and the quantity of the functional group, but also the position of functionalization on the base molecules is decisive for the redox potentials^26^.

The calibration equation for the prediction of redox potentials versus SHE, using the PBE_s_ (≡ T) calculated reaction energies is:

1 $${E}^{\mathrm{o}}=-0.409[\Delta{E}_{\mathrm{rxn}}^{\mathrm{DFT}}]-0.193$$

The performance metrics of all the DFT functionals are given in Supplementary Table S2.

### Comparison of low-level methods: FF, SEQM and DFTB

**Figure 3:**
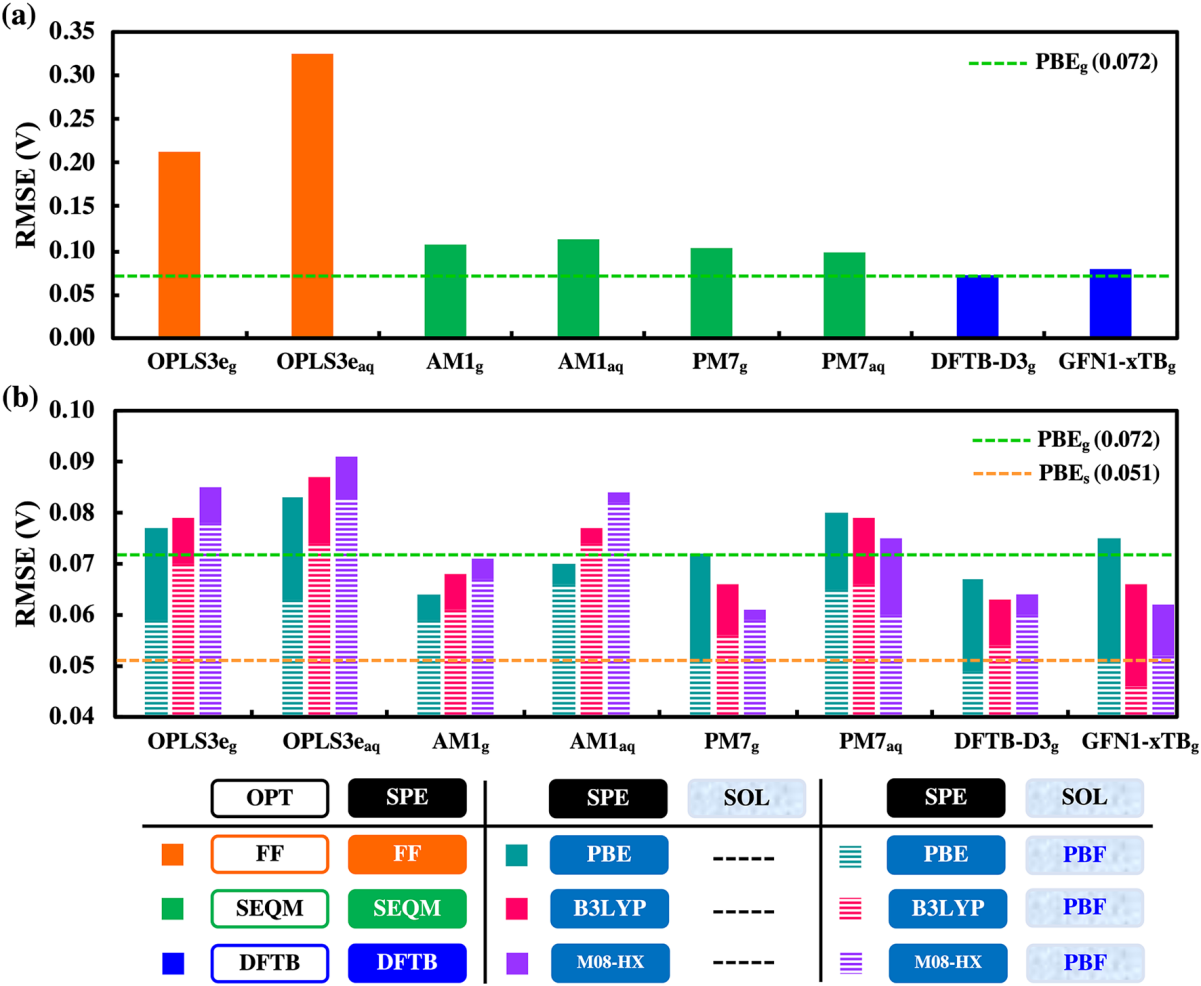
Performance comparisons of low-level methods: FF, SEQM and DFTB. (**a**) RMSE of SPE data calculated at the three different levels of theory. Similarly, (**b**) RMSE of DFT calculated SPE data on the geometries obtained from the three different levels of theory. In (**b**), the solid bars show SPE results without implicit solvation effects, whereas the dashed bars show results with implicit solvation effects taken into account. The dashed green horizontal line represents PBE_g_ (R^2^ = 0.954, RMSE = 0.072 V) benchmark and the dashed orange horizontal line represents PBE_s_ (R^2^ = 0.977, RMSE = 0.051 V) benchmark.

After establishing the effectiveness of DFT-based methods, we next consider the computationally less costly methods for the optimization of geometries and the prediction of energies. As summarized in Fig. 3 and Supplementary Fig. S4, we employ different lower level methods such as FF, SEQM and DFTB for geometry optimizations. To obtain $\Delta{E}_{\mathrm{rxn}}^{\mathrm{T}}$ of the reactions, we use the optimized structures of compounds and perform SPE calculations via the following three schemes:

I. SPE values are taken directly from low-level methods after the geometry optimizations in gas- or aqueous-phases. Aqueous-phase optimizations are performed only by using FF and SEQM (but not DFTB, as explained in Computational Workflow).
II. SPE values are taken from gas-phase DFT calculations using three different functionals (PBE, B3LYP and M08-HX), on the molecular geometries obtained via scheme (I).
III. SPE values are taken from DFT calculations with implicit solvation using three different functionals (PBE, B3LYP and M08-HX), on the molecular geometries obtained via scheme (I).

Several observations are made by comparing the R^2^ and RMSE data across the various combinations of methods.

### Comparisons within scheme (I)

When comparing redox potential predictions from SPE data of scheme (I) to PBE_g_ benchmark (R^2^ = 0.954, RMSE = 0.072 V), we make the following observations (note: subscript ‘g’ represents gas-phase and subscript ‘aq’ represents aqueous-phase geometry optimizations at a given level of theory):

- In Fig. 3a, gas-phase (OPLS3e_g_: R^2^ = 0.596, RMSE = 0.213 V) and aqueous-phase (OPLS3e_aq_: R^2^ = 0.060, RMSE = 0.325 V) calculated FF SPEs are significantly worse than PBE_g_. Just as observed for DFT methods, aqueous-phase FF optimizations yield worse results than their gas-phase counterpart. Clearly, the internal energy predictions at the FF level are inaccurate.
- In Fig. 3a, gas-phase SEQM methods show significantly better performance compared to FF method, and are close to PBE_g_ benchmark. Of note are the AM1_g_ (R^2^ = 0.899, RMSE = 0.107 V) and PM7_g_ (R^2^ = 0.906, RMSE = 0.103 V) methods. The aqueous-phase SEQM geometry optimizations with COSMO solvation model result in very similar prediction accuracies to their gas-phase counterparts for both AM1_aq_ (R^2^ = 0.886, RMSE = 0.113 V) and PM7_aq_ (R^2^ = 0.915, RMSE = 0.098 V).
- In Fig. 3a, gas-phase DFTB methods perform as good as PBE_g_ benchmark, with parameter sets DFTB-D3_g_ (R^2^ = 0.953, RMSE = 0.072 V) and GFN1-XTB_g_ (R^2^ = 0.944, RMSE = 0.079 V).

### Comparisons within scheme (II)

When comparing predictions from SPE data of scheme (II) to PBE_g_ benchmark (R^2^ = 0.954, RMSE = 0.072 V), we make the following observations:

- In Fig. 3b (solid bars), the performances of gas-phase DFT calculations of SPEs on gas-phase FF geometries (OPLS3e_g_: R^2^ = 0.947, RMSE = 0.077 V) are significantly better than their counterparts from scheme (I). The same is also observed for gas-phase DFT calculations of SPEs on aqueous-phase FF geometries (OPLS3e_aq_: R^2^ = 0.939, RMSE = 0.083 V). However, even after performing DFT calculations of SPEs, the OPLS3e_aq_ performs worse than OPLS3e_g._
- In Fig. 3b (solid bars), gas-phase DFT calculations of SPEs on gas-phase SEQM geometries also show improved prediction accuracies with respect to their counterparts from scheme (I). The two best SEQM methods are AM1_g_ (R^2^ = 0.963, RMSE = 0.064 V) and PM7_g_ (R^2^ = 0.954, RMSE = 0.072 V), with performances equivalent to PBE_g_ benchmark. Gas-phase DFT calculations of SPE on aqueous-phase SEQM geometries resulted in worse predictions for both AM1_aq_ (R^2^ = 0.956, RMSE = 0.070 V) and PM7_aq_ (R^2^ = 0.943, RMSE = 0.080 V), though they are still better with respect to their counterparts from scheme (I).
- In Fig. 3b (solid bars), gas-phase DFT calculations of SPEs on gas-phase DFTB geometries also show slightly improved prediction accuracies and are slightly better than PBE_g_ benchmark, with parameter sets DFTB-D3_g_ (R^2^ = 0.960, RMSE = 0.067 V) and GFN1-XTB_g_ (R^2^ = 0.949, RMSE = 0.075 V).

### Comparisons within scheme (III)

Upon including implicit solvation effects during DFT calculations of SPE, the performances of low-level methods versus the corresponding PBE_s_ benchmark (R^2^ = 0.977, RMSE = 0.051 V) can be described as follows (please note that the subscript ‘s’ represents gas-phase geometry optimization but with implicit solvation included while calculating SPE with DFT):

- In Fig. 3b (dashed bars), RMSEs are lowered by 0.02 V for FF optimized geometries, both from gas- (OPLS3e_g_: R^2^ = 0.969, RMSE = 0.059 V) and aqueous-phases (OPLS3e_aq_: R^2^ = 0.964, RMSE = 0.063 V), in comparison to their counterparts from scheme (II). Surprisingly, the performances of these methods are close to PBE_s_ benchmark. This shows that even though the thermochemistry with FF obtained energies is not accurate (as observed in Scheme I), the quinone molecule geometries from FF are good enough for performing DFT SPE calculations.
- In Fig. 3b (dashed bars), prediction accuracies from SEQM optimized geometries are improved when compared to their counterparts in scheme (II). The two best SEQM methods are AM1_g_ (R^2^ = 0.969, RMSE = 0.059 V) and PM7_g_ (R^2^ = 0.976, RMSE = 0.051 V). Yet again, predictions from aqueous-phase SEQM geometries resulted in slightly worse results for both AM1_aq_ (R^2^ = 0.961, RMSE = 0.066 V) and PM7_aq_ (R^2^ = 0.962, RMSE = 0.065 V). Interestingly, the performances of these SEQM methods are close to PBE_s_ benchmark.
- In Fig. 3b (dashed bars), prediction accuracies from DFTB optimized geometries improve when compared to their counterparts in scheme (II). Strikingly, both sets of DFTB parameters, DFTB-D3_g_ (R^2^ = 0.978, RMSE = 0.049 V) and GFN1-XTB_g_ (R^2^ = 0.977, RMSE = 0.051 V), perform better than PBE_s_ benchmark.

## Discussion

All the variations in computational methods that are used for geometry optimizations and SPE calculations, also with and without implicit solvation effects, are found to influence the prediction accuracies to varying degrees. First, for all methods, gas-phase DFT calculations of SPEs lead to significant improvements in prediction accuracies. According to these results, computationally demanding DFT geometry optimizations are hardly necessary for a first-order screening of large numbers of candidate molecules. Instead, either of SEQM or DFTB methods may be employed for the task of gas-phase geometry optimizations. Secondly, SPE calculations employing the PBE functional are generally better performing than SPEs obtained from computationally more costly B3LYP and M08-HX functionals. Thirdly, for all low-level methods, the inclusion of implicit solvation during DFT calculations of SPEs leads to improved prediction accuracies. Finally, the results confirm that the effects of geometry optimizations in aqueous-phase are minimal and they often result in slightly worse prediction accuracies.

The calibration equation for the prediction of redox potentials versus SHE, using the DFTB-D3_g_ (≡ T) calculated reaction energies is:

2 $${E}^{\mathrm{o}}=-0.447[\Delta{E}_{\mathrm{rxn}}^{\mathrm{DFTB}}]-0.823$$

The performance metrics of all the low-level methods, as well as their combinations with high-level methods, are given in Supplementary Tables S3-S9. It is worth pointing out that a recent study by Tabor et al.^27^ also considered DFT and SEQM based methods for performing a similar calibration exercise to determine an optimum method for predicting redox potentials of 28 quinone molecules using $\Delta{E}_{\mathrm{rxn}}$ as a descriptor. In their study, at the DFT level, they considered the B3LYP functional with 6-311+G(d,p) basis set, which is very similar to the basis set used in this study. At the SEQM level, they considered the PM7 method. The MAD with gas-phase DFT was 0.056 V (vs. 0.058 V using PBE in this work) and with implicit aqueous solvation using the PCM model was 0.041 V (vs. 0.038 V using PBE with PBF solvation model in this work). When using the PM7 method, their predicted MAD value was 0.052 V in the gas phase (vs. 0.065 V in this work), and 0.067 V with the COSMO solvation model (vs. 0.067 V in this work). The difference between the results from the two studies are quite small and vary mostly in the third decimal place, which is not surprising given the common pool of molecules. For predicting redox potentials, we recommend using Eq. 1 not only because it has slightly better prediction accuracy but also because it covers a larger group of experimented molecules with a wider range of redox potentials.

### Accuracy of predictions versus cost of calculations

**Figure 4:**
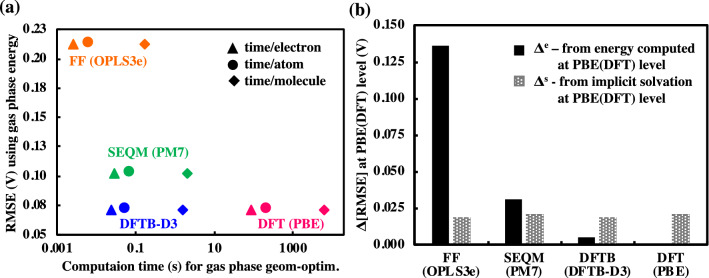
(**a**) Variation of computation time for gas-phase geometry optimizations versus the corresponding RMSEs from the four representative methods of each level of theory, namely OPLS3e (FF), PM7 (SEQM), DFTB-D3 (DFTB), and PBE (DFT). The SPEs are taken directly after optimization runs at corresponding levels of theory. A logarithmic scale is used when plotting the computation time. (**b**) Bar plot for change in RMSE values, Δ[RMSE], due to gas-phase DFT calculation of SPE and inclusion of implicit solvation. Solid bars, Δ^e^, show the impact of DFT calculation of SPEs on geometries obtained from lower level methods. Dashed bars, Δ^s^, show the impact of implicit solvation on the DFT calculated SPEs.

In addition to determining accurate methods for predicting the redox potentials of electroactive quinones, a major aim of the current study is to decide on the methods that are most suited for both standalone and HTCS studies for which a balance between the speed of computations and the accuracy of results is desireable. This is especially important when the robust DFT calculations become impractical for studies on a vast chemical space (10^3^ ~ 10^6^ compounds). Thus, an estimation of trade-offs between the computational accuracy and its cost is useful for efficient screening studies. For a comparison of the computation time, we selected a representative method for each level of theory, namely, OPLS3e (FF), PM7 (SEQM), DFTB-D3 (DFTB) and PBE (DFT). Next, noting that the geometry optimizations are usually the most computationally demanding processes, we optimized the geometries of all the molecules in gas-phase using these representative methods. We added the FF geometry optimization time to all other methods’ calculation times, since we use it as the base method for performing all other geometry optimizations (as explained in Computational Workflow). An averaged computation time, as obtained from five different runs that actually showed no significant variation, is used to describe the relation between RMSEs versus the computational cost. As shown in Fig. 4a, DFTB-D3 is almost as accurate as DFT in predicting the redox potentials, while it requires substantially less (~ 10^3^ times) computing time. Also noting that DFTB-D3 has previously been applied for calculations of large systems at relatively lower computational costs and with similar accuracies to that of the higher level (i.e. DFT-GGA) methods^41^, we suggest that for the small quinone redox compounds the DFTB-D3 method provides a good compromise between prediction accuracy and computational cost. Accordingly, for HTCS studies that are aimed to work on extremely large chemical spaces of molecules, we suggest DFTB-D3 computations on OPLS3e optimized geometries, which have an RMSE of 0.072 V that is close to the RMSE of 0.051 V of the DFT (PBE) calculations, as a way to accelerate the virtual screening of compounds.

### Effects of geometry optimizations at various levels of theory and implicit solvation

For the set of methods considered in the previous section, we also quantify the effect of gas-phase DFT calculation of SPEs by using the geometries that have been obtained via low-level methods. As shown in Fig. 4b (solid bars), the improvement in prediction accuracy, Δ^e^ RMSE, is most significant for geometries optimized by OPLS3e (Δ^e^ RMSE = 0.136 V), followed by PM7 (Δ^e^ RMSE = 0.031 V), and then by DFTB-D3 (Δ^e^ RMSE = 0.005 V). These results show that SEQM, and more pronouncedly, DFTB methods do not only predict the reaction energies accurately, but they also predict the geometries of the compounds as comparable to that of DFT. For the same set of methods, we also investigate relationships between the differences in molecular geometries and the calculated values of Δ^e^ RMSE. By performing structure superposition analysis we compared the optimized geometries from different methods to the reference, gas-phase PBE optimized geometries. The average root-mean-square deviation (RMSD) of all the 86 reactant and product molecules under various atomic constraints are shown in Table 1. First, under all constraints OPLS3e has the largest average RMSD with respect to PBE, which is expected. Secondly, when considering all atoms or only heavy atoms, PM7 and DFTB-D3 are very similar in geometrical difference with regards to PBE. Thirdly, it is surprising to note that DFTB-D3, while the most accurate of the low-level methods, does not necessarily provide the geometry closest to PBE (i.e. lowest average RMSD) when all atoms or non-hydrogen atoms are considered. However, when considering only the carbon atoms in the ring structure of molecules, DFTB-D3 produces structures that are closest to PBE. Given that the cyclic carbon atoms are a large fraction of the total number of atoms, it is possible that being able to represent the geometry of the rings accurately is what gives DFTB-D3 (Δ^e^ RMSE = 0.005 V) an advantage over PM7 (Δ^e^ RMSE = 0.031 V) and OPLS3e (Δ^e^ RMSE = 0.136 V) for the redox potential predictions.

**Table 1:** The difference in the optimized molecular geometry using various calculation methods with reference to PBE (DFT) geometry under various atomic constraints.

Method	All atoms	Non-hydrogen	Only ring carbon
OPLS3e (FF)	0.148	0.120	0.052
PM7 (SEQM)	0.132	0.098	0.050
DFTB-D3 (DFTB)	0.135	0.102	0.043

The average RMSD values for the 86 molecules are shown in units of Å. RMSD values have been calculated for all atoms, all non-hydrogen atoms, and carbon atoms of molecule rings.

Next, we quantify the improvements in prediction capability of the methods, with respect to gas-phase DFT calculation of SPE, due to the inclusion of implicit solvation effects, Δ^s^ RMSE (dashed bars). As shown in Fig. 4b, an improvement in prediction accuracy is evident for all levels of theory and Δ^s^ RMSE are similar at each level of theory. The decrease in RMSEs are 0.018, 0.021, 0.018, and 0.021 V for OPLS3e, PM7, DFTB-D3, and PBE, respectively. These results show that the amount of improvement due to the inclusion of implicit solvation is independent of the source of geometry. These findings are useful, for instance, when building machine learning (ML) models for the prediction of solvation energies directly from simple cheminformatics-based descriptors without a real need for an explicit knowledge of compound geometries.

In addition to the above findings, the results presented here are expected to be useful for generating accurate and large quantity chemical data on compounds, which can later be utilized by data-driven machine learning models for expanding the boundaries of search space during candidate compound explorations. Firstly, as has been argued in recent studies^27,29^, using the computationally costly quantum chemical calculations for millions of molecules, which is required for building powerful ML models, is still a major bottleneck. One of the key findings of this work is that the DFTB method is nearly as accurate as DFT when it comes to the prediction of quinone redox potentials. Thus, the data scarcity bottleneck can be addressed directly by using DFTB to generate large quantities of reliable reference data. Secondly, it has been demonstrated quantitatively in this work that the improvement due to the use of quantum chemistry methods, when compared to SEQM and DFTB methods, corresponds to an energy contribution that constitutes only a minor fraction of the total energy. An increasingly popular strategy that utilizes this fact for the property predictions at quantum chemical accuracy is Δ-ML^42^. In this strategy, by training on quantum chemical reference data, a ML model is trained to predict the correction terms, Δ, to values that have been computed using the inexpensive methods. This way, instead of performing quantum chemical calculations directly on all candidate compounds, the energy values are interpolated from a selected inexpensive baseline theory to quantum chemical accuracy. In our study we recommend a choice of methods, for both reference (e.g. M08-HX) and base line (e.g. DFTB-D3) data, to perform such Δ-ML tasks. Finally, we would like to emphasize again, that this work provides a general methodology for determining the optimum combination of methods with an example case of the quinone family of compounds. This knowledge is directly relevant for developing advanced decision-making frameworks that can automatically decide on the most optimum combination of computational methods for a target class of chemical compounds.

## Methods

### Thermodynamic principle

The reaction energy difference between the reactant and product is used as an approximation for the Gibbs free energy of proton-coupled electron transfer redox reactions. During a redox reaction in the aqueous-phase:

3 $$\mathrm{Q}+{2\mathrm{H}}^{+}{+ 2e}^{- }\to {\mathrm{QH}}_{2}$$

The hydroquinone, QH_2_, compounds can be generated from the quinone, Q, compounds via a two-electron two-proton redox reaction^22^. A quantitative measure of the favorability of a given reaction is the change in standard Gibbs free energy, $\Delta{G}_{\mathrm{rxn}}^{\mathrm{o}}$. According to the Nernst equation, the equilibrium potential of a redox reaction, ${E}^{\mathrm{o}}$, is related to the change in the standard Gibbs free energy per coulomb of charge transferred during the electrochemical reaction as:

4 $${E}^{\mathrm{o}}=- \Delta {G}_{\mathrm{rxn}}^{\mathrm{o}}/nF$$

where *n* = 2 is the number of electrons and *F* is the Faraday constant. Typically, ${E}^{\mathrm{o}}$ is measured relative to the Standard Hydrogen Electrode (SHE) and $\Delta{G}_{\mathrm{rxn}}^{\mathrm{o}}$ is computed at standard conditions. To calculate $\Delta{G}_{\mathrm{rxn}}^{\mathrm{o}}$ we use the following equation:

5 $$\Delta {G}_{\mathrm{rxn}}^{\mathrm{o}}= \Delta {G}^{\mathrm{o}}\left({\mathrm{QH}}_{2}\right)-[{G}^{\mathrm{o}}(\mathrm{Q})+{G}^{\mathrm{o}}({\mathrm{H}}_{2})]$$

in which $\Delta{G}_{\mathrm{rxn}}^{\mathrm{o}}$ is expressed simply as the difference in the standard free energies of the reactants and products. Thermodynamically, $\Delta{G}_{\mathrm{rxn}}^{\mathrm{o}}$ can be described as a sum of contributions arising from the change in internal energy ($\Delta{U}_{\mathrm{rxn}}$), pressure–volume ($p\Delta{V}_{\mathrm{rxn}}$) and entropic ($T\Delta{S}_{\mathrm{rxn}}$) contributions due to reaction as:

6 $$\Delta {G}_{\mathrm{rxn}}^{\mathrm{o}}= \Delta {U}_{\mathrm{rxn}}+{p \Delta V}_{\mathrm{rxn}}-{T \Delta S}_{\mathrm{rxn}}$$

The change in internal energy can be further decomposed as $\Delta{U}_{\mathrm{rxn}}= \Delta {E}_{\mathrm{rxn}}+\Delta\mathrm{ZPE}$, where $\Delta{E}_{\mathrm{rxn}}$ is the reaction energy and $\Delta\mathrm{ZPE}$ is the change in zero-point energy. In the present work, the ZPE contributions to the internal energy, changes in pressure–volume, and entropic contributions are neglected, thus effectively using the approximation:

7 $$\Delta {G}_{\mathrm{rxn}}^{\mathrm{o}} \cong \Delta {E}_{\mathrm{rxn}}={E}^{\mathrm{aq}}\left({\mathrm{QH}}_{2}\right)-\left[{E}^{\mathrm{aq}}\left(\mathrm{Q}\right)+{E}^{\mathrm{aq}}\left({\mathrm{H}}_{2}\right)\right]$$

where ${E}^{\mathrm{aq}}$ represents the theoretically calculated internal energy of species in aqueous-phase. All the other contributions are ignored because they require extra calculation steps, computational resources, and thus, are not suitable from the perspective of HTCS. Nevertheless, the effects of ignoring these other contributions on the accuracy of predictions are discussed in the main text. In this work, the descriptor of choice, $\Delta{E}_{\mathrm{rxn}}$, is calculated with the inclusion of aqueous solvation effects, which requires additional computation time. To quantify the effect of solvation on prediction accuracy, another approximation is considered by ignoring solvation such that Eq. (7) can be rewritten using internal energies calculated in the gas-phase as:

8 $$\Delta {G}_{\mathrm{rxn}}^{\mathrm{o}} \cong \Delta {E}_{\mathrm{rxn}}={E}^{\mathrm{g}}\left({\mathrm{QH}}_{2}\right)-\left[{E}^{\mathrm{g}}\left(\mathrm{Q}\right)+{E}^{\mathrm{g}}\left({\mathrm{H}}_{2}\right)\right]$$

Under these set of approximations, the calculated change in internal energies $\Delta{E}_{\mathrm{rxn}}$ from Eqs. (7) and (8) are linked to the measured redox potentials using Eq. (4). We used various theoretical methods, as explained below in computational details, to calculate $\Delta{E}_{\mathrm{rxn}}$, and discuss their performance for the prediction of experimentally measured redox potentials.

### Computational details

We developed a systematic computational approach involving one FF, nine SEQM, two DFTB, and eleven DFT methods, as well as their combination with implicit solvation environments, to predict the experimentally measured redox potentials of quinone-based electroactive. The MacroModel program is used for FF configurational searches and geometry optimizations, while the Jaguar program^38^ is used for DFT calculations, all as implemented in the Schrödinger Materials Science Suite (version 2019-2). MOPAC and DFTB calculations are performed using the ADF program^43^. Molecular structures are optimized both in gas- and aqueous-phase using the OPLS3e FF, which provides a broad coverage of small compounds^36^. The gas-phase FF optimized geometries are used as inputs to perform gas- and aqueous-phase geometry optimizations using nine different SEQM methods, including AM1^44^, MNDO^45^, MNDOD^46^, PM3^47^, PM6^48^, PM6-D3^49^, PM6-D3H4X^34^, PM7^50^ and RM1^51^. The aqueous-phase geometry optimizations at the SEQM level are performed using the COSMO-RS solvation model^52,53^. The choice of this solvation method is constrained by the present availability in the ADF program. The gas-phase FF optimized geometries are also used as inputs for DFTB optimizations using the DFTB-D3^54^ and GFN1-xTB^55,56^ methods. The DFTB-D3 computations are performed with a self-consistent charge cycle using the QuasiNANO-2015 parameter set^33^, while the parameters for GFN1-xTB are taken from the work of Grimme et al.^55,56^ The aqueous-phase geometry optimizations of molecules are not performed with the DFTB method, since currently there is no available routine for this task in the ADF program. Finally, FF minimized geometries are used as inputs to perform geometry optimizations in gas-phase DFT calculations using different flavors of exchange–correlation functionals, including the local density approximation (LDA)^57^, generalized gradient approximation (GGA)^58^, hybrid and meta-GGA functionals^58^, all of which vary drastically in their accounting of the exchange–correlation energy. For the geometries that have been obtained from FF, SEQM and DFTB optimizations, the DFT level SPEs are computed in gas-phase, and subsequently in aqueous-phase using only the PBE, B3LYP, and M08-HX functionals, as they are well accepted in the community but also span a wide range of ways for accounting for the exchange–correlation effects^58^. A total of 11 exchange–correlation functionals, also including some of the D3 dispersion^59,60^ corrected variants, are used for high-level OPT and SPE calculations. These functionals include LDA^57^, PBE^40,61^, PBE-D3^59^, BLYP^62^, BLYP-D3^59^, B3LYP^62,63^, B3LYP-D3^59^, PBE0^64^, PBE0-D3^59^, HSE06^65^, and M08-HX^39^. Due to computational costs, the DFT aqueous-phase geometry optimizations are performed only with the following functionals: PBE, B3LYP, and M08-HX.

As DFT options in Jaguar, we chose “medium” grid density for OPT and “fine” grid density for SPE calculations. Energy and RMS density matrix change convergence criteria are set to the default values of 5.0 $\times$ 10^–5^ and 5.0 $\times$ 10^–6^ Hartree, respectively. The default direct inversion in the iterative subspace is employed as the convergence scheme. For OPT, Jaguar’s mixed pseudospectral grids with default cutoffs are employed. For SPE calculations, we used pseudospectral grids with accurate cutoffs. To treat solvated molecules in water, we used the standard PBF solver with water as the solvent^37,38^. The calculations are performed with LACVP^**++^ basis set with polarization and diffuse functions^66,67^. The LACVP basis set is chosen here because it includes an effective core potential (ECP), which represents the effect of the core electrons in a parametrized form. The use of ECPs speeds up calculations on compounds that contain heavy elements. For the elements from H to Ar, LACVP and the widely employed 6-31G are essentially indistinguishable when evaluating the ground-state properties. The quinone molecules considered in this work contain the elements of C, H, O, N, S, F and Cl, and thus, the use of LACVP^**++^ basis set in this work is consistent with the use of 6-31G^**++^ basis set.

### Calibration data and performance metrics

We collected redox potential data from 43 quinone redox couples in acidic aqueous solution^16,19,27,68^. In consideration of prediction accuracy and universality for the calibration models, the selection of available experimental data has been expanded within various quinone molecules, rather than using monotonous structural patterns. This is because compounds decorated with chemical functional groups usually show different redox properties as well as charge/discharge capacities when compared to their undecorated counterparts. The experimental molecules cover both quinone cores and their functionalized derivatives with multiple substituted groups including –SO_3_H, –COOH, –OH, –CH_3_, –F and –Cl (see Supplementary Table S1) and the redox data spans a broad range experimental redox potentials between −0.084 and 1.21 V. The redox couples are chosen consistently from measurements that were performed under similar experimental conditions, such as *T* = 298.15 K, pH = 0, and highly conducting salts. The correlations between experiments and calculations are expressed in terms of two commonly used coefficients, namely the coefficient of determination (R^2^) and root-mean-square error (RMSE). R^2^ and RMSE are calculated using the definitions from the Originlab. All benchmark simulations of calculation time have been performed on a single core of Intel Core i9-9960X 3.10 GHz CPU with Ubuntu 18.04 Bionic Beaver as the operating system.

## Supplementary Information

Supplementary Information

## Data Availability

The generated computational data of compounds is provided in Supplementary Table S1.
