# Supplementary material for: Comparison of computational chemistry methods for the discovery of quinone-based electroactive compounds for energy storage
Source: Sci Rep. 2020 Dec 17;10:22149. doi: 10.1038/s41598-020-79153-w (PMC7746720; doi:10.1038/s41598-020-79153-w)
Supplement: Supplementary file 1 — Supplementary Information [file 41598_2020_79153_MOESM1_ESM.pdf]

Supplementary Information

Comparison of computational chemistry methods for the discovery of quinone-based electroactive compounds for energy storage

Qi Zhang[1,2,3]*, Abhishek Khetan*[1,2]*, Süleyman Er*[1,2*]

[1] DIFFER – Dutch Institute for Fundamental Energy Research, De Zaale 20, 5612 AJ Eindhoven, The Netherlands

[2] CCER – Center for Computational Energy Research, De Zaale 20, 5612 AJ Eindhoven, The Netherlands

[3] Department of Applied Physics, Eindhoven University of Technology, 5600 MB Eindhoven, The Netherlands.

[*] E-mail: s.er@differ.nl

Explanation of the abbreviations used in the below tables:

SMILES: simplified molecular-input line-entry system

DFT: density functional theory

SEQM: semi-empirical quantum mechanics

DFTB: density functional tight-binding

FF: Force Field

OPT: geometry optimization

SPE: single-point energy

SOL : implicit aqueous medium

R^2: the coefficient of fit

RMSE : the root mean squared error

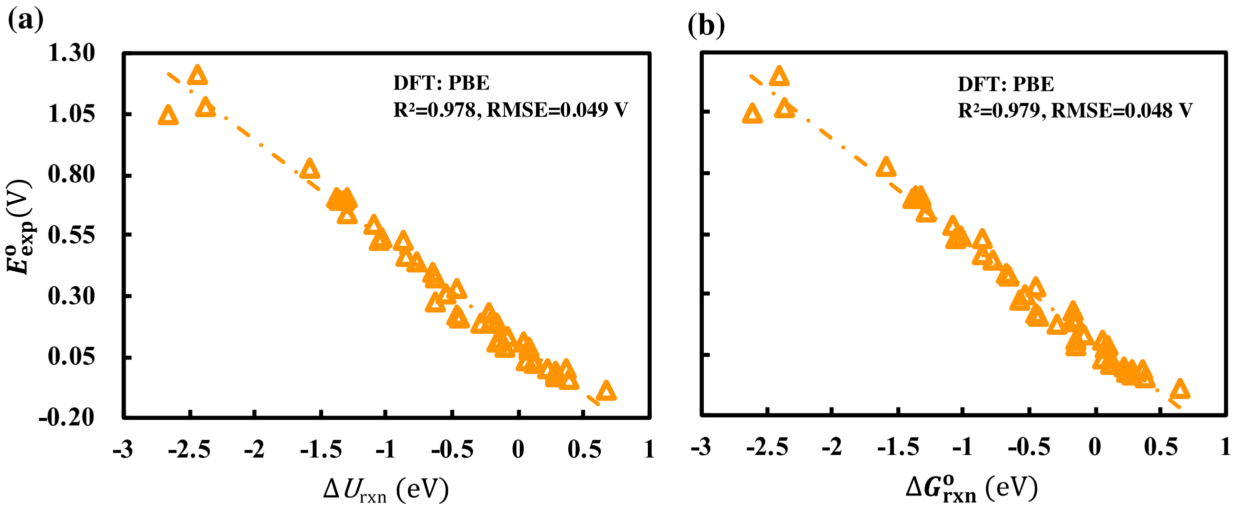

Figure S1. Performance of (a) ΔU_{rxn} and (b) ΔG^{o}_{rxn} descriptors for the prediction of experimentally measured redox potentials, E^{o}_{exp}. The DFT calculations were performed using the PBE functional.

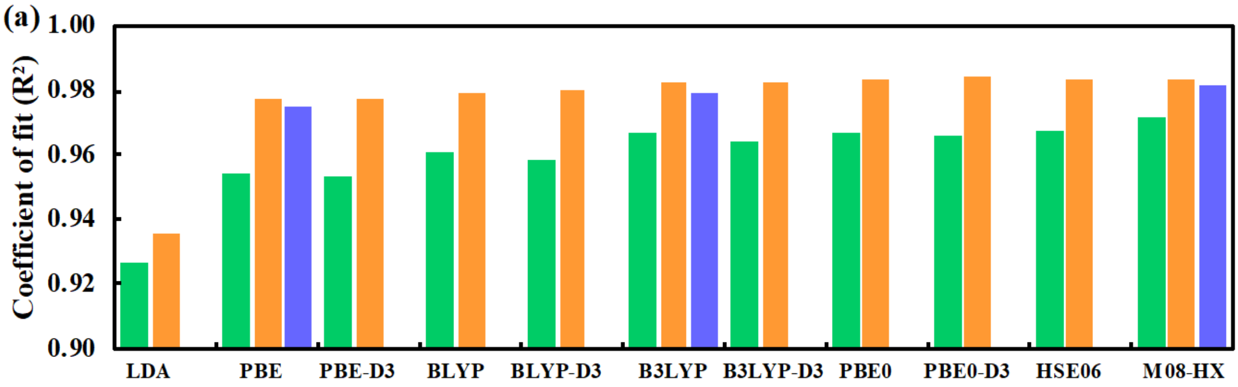

Figure S2. Performance comparisons of different DFT exchange-correlation functionals for the prediction of experimentally measured redox potentials, E^{o}_{exp}. The bar plot shows R^2 values for all the functionals considered in this work. The color green represents both OPT and SPE in gas-phase, the color orange represents OPT in gas-phase followed by SPE with SOL (i.e., including implicit aqueous solvation), and color blue represents both OPT and SPE with SOL.

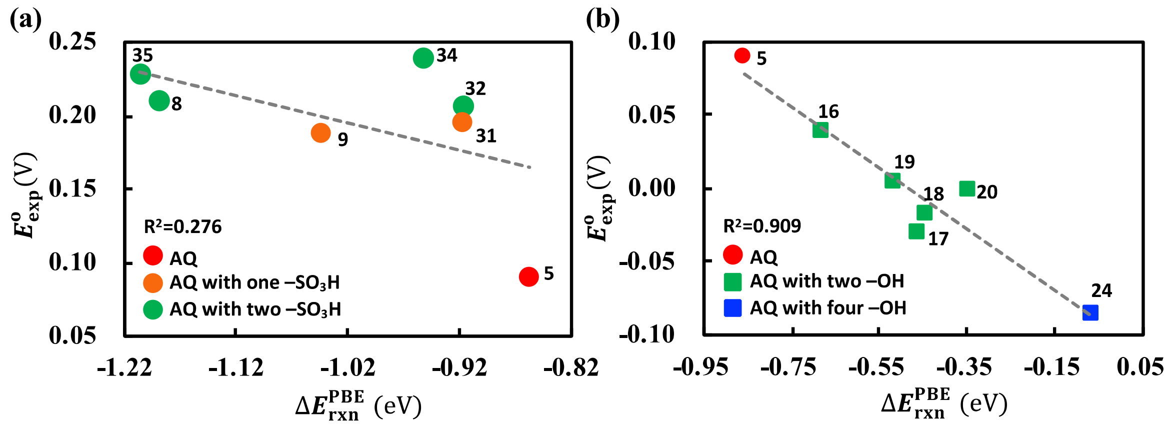

Figure S3. The effects of (a) –SO3H and (b) –OH chemical functionalization groups on the redox potentials of anthraquinone-derivatives.

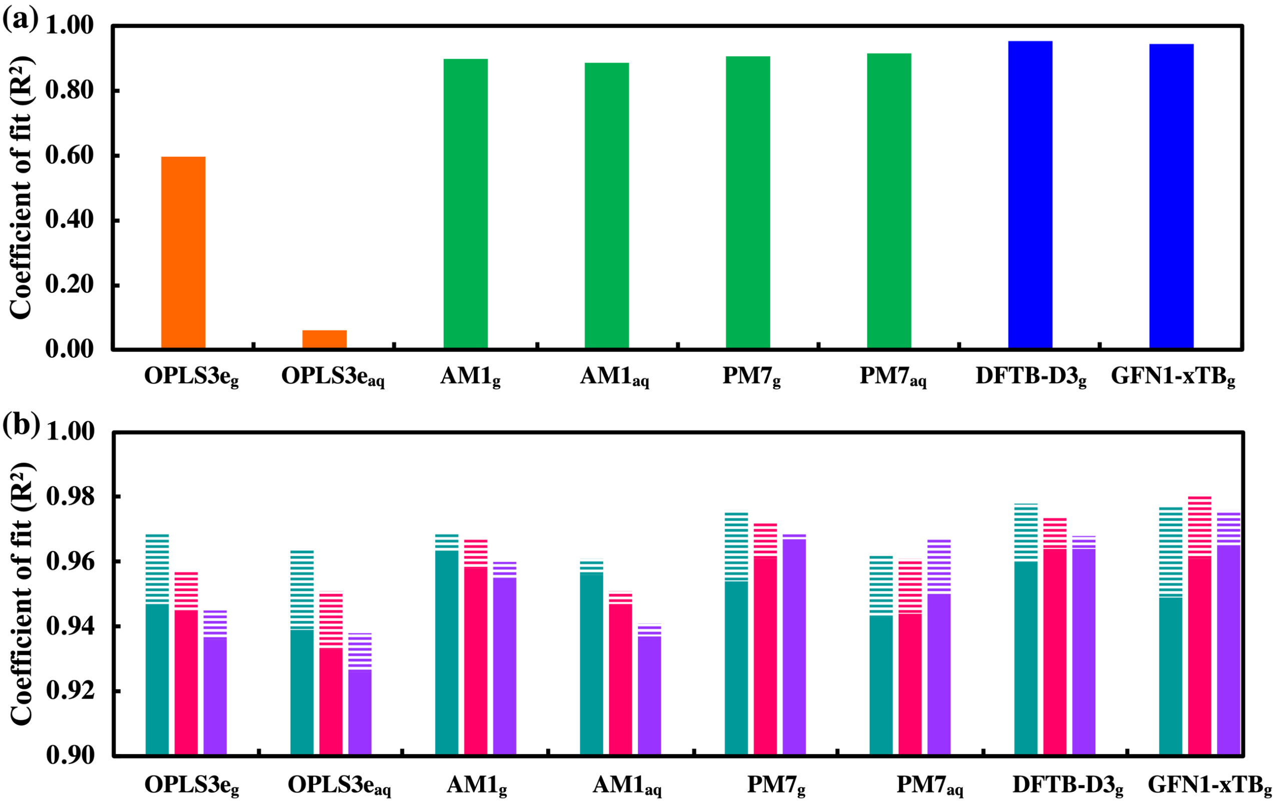

Figure S4. Performance comparisons of low-level methods: FF, SEQM and DFTB. (a) shows R^2 for SPEs calculated at these three different levels of theory. Similarly, (b) shows R^2 for DFT calculated SPE data on the geometries obtained from these three different levels of theory. In (b), the solid bars show SPE results without the implicit solvation effect, whereas the dashed bars show the results with the implicit solvation effects taken into account.

Table S1. A summary of 2D structures, SMILES representations, experimental and predicted redox potentials (V versus SHE) of the compounds.

#	Molecule	Smiles	E^{o}_{exp}	$E^{o}_{pre\_g}$	$E^{o}_{pre\_s}$	$E^{o}_{pre\_aq}$	Reference
**1**	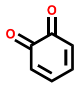	O=C1C(=O)C=CC=C1	0.831	0.768	0.764	0.765	[4]
**2**	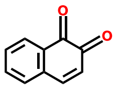	O=C1C(=O)C=Cc(c12)cccc2	0.547	0.574	0.535	0.531	[4]
**3**	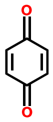	O=C1C=CC(=O)C=C1	0.699	0.617	0.671	0.673	[4]
**4**	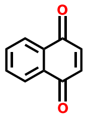	O=C1C=CC(=O)c(c12)cccc2	0.470	0.436	0.462	0.460	[4]
**5**	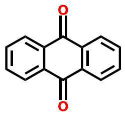	c1cccc(c12)C(=O)c3c(C2=O)cccc3	0.090	0.178	0.158	0.155	[4]
**6**	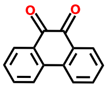	c1cccc(c12)c3c(C(=O)C2=O)cccc3	0.442	0.477	0.420	0.418	[4]
**7**	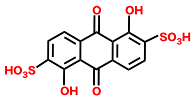	O=S(=O)(O)c(c1O)ccc(c12)C(=O)c3c(C2=O)ccc(c3O)S(=O)(=O)O	0.116	0.246	0.185	0.154	[3]
**8**	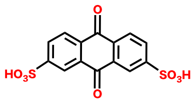	c1cc(S(=O)(=O)O)cc(c12)C(=O)c3c(C2=O)ccc(c3)S(=O)(=O)O	0.21	0.280	0.294	0.253	[1]
**9**	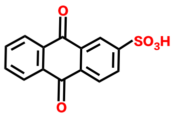	c1cccc(C2=O)c1C(=O)c(c23)cc(cc3)S(=O)(=O)O	0.187	0.227	0.234	0.211	[1]

**10**	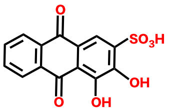	c1cccc(C2=O)c1C(=O)c(c23)cc(S(=O)(=O)O)c(O)c3O	0.082	0.070	0.076	0.091	[1]
**11**	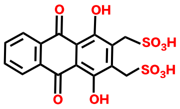	c1cccc(C2=O)c1C(=O)c(c23)c(O)c(CS(=O)(=O)O)c(c3O)CS(=O)(=O)O	0.02	0.061	0.046	0.053	[1]
**12**	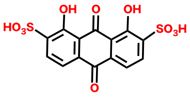	c1cc(S(=O)(=O)O)c(O)c(c12)C(=O)c3c(C2=O)ccc(c3O)S(=O)(=O)O	0.12	0.192	0.176	0.184	[1]
**13**	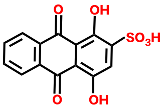	Oc1c(S(=O)(=O)O)cc(O)c(c12)C(=O)c3c(C2=O)cccc3	0.09	0.076	0.074	0.055	[3]
**14**	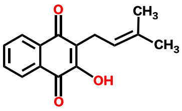	c1cccc(c12)C(=O)C(=C(C2=O)O)CC=C(C)C	0.333	0.297	0.291	0.306	[2]
**15**	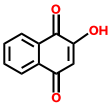	c1cccc(c12)C(=O)C(O)=CC2=O	0.308	0.274	0.333	0.332	[2]
**16**	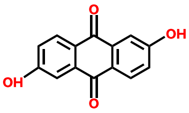	c1c(O)ccc(c12)C(=O)c3c(C2=O)ccc(c3)O	0.039	0.122	0.087	0.088	[2]
**17**	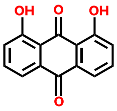	c1ccc(O)c(c12)C(=O)c3c(C2=O)cccc3O	-0.03	-0.016	-0.003	-0.004	[2]
**18**	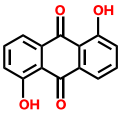	Oc1cccc(c12)C(=O)c3c(C2=O)cccc3O	-0.017	-0.046	-0.010	-0.010	[2]

**19**	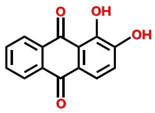	Oc1c(O)ccc(c12)C(=O)c3c(C2=O)cccc3	0.005	0.015	0.020	0.017	[2]
**20**	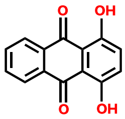	Oc1ccc(O)c(c12)C(=O)c3c(C2=O)cccc3	-0.001	-0.074	-0.049	-0.046	[2]
**21**	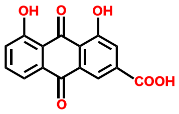	c1ccc(O)c(c12)C(=O)c3c(C2=O)cc(C(=O)O)cc3O	0.041	0.011	0.055	0.059	[2]
**22**	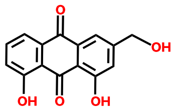	c1ccc(O)c(c12)C(=O)c3c(C2=O)cc(CO)cc3O	-0.008	-0.025	-0.008	-0.039	[2]
**23**	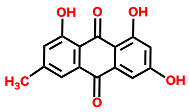	c1c(C)cc(O)c(c12)C(=O)c3c(C2=O)cc(O)cc3O	-0.040	-0.061	-0.047	-0.041	[2]
**24**	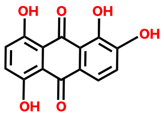	Oc1ccc(O)c(c12)C(=O)c3c(C2=O)ccc(O)c3O	-0.084	-0.197	-0.165	-0.165	[2]
**25**	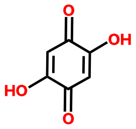	OC1=CC(=O)C(O)=CC1=O	0.382	0.264	0.364	0.362	[2]
**26**	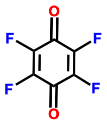	O=C1C(F)=C(F)C(=O)C(F)=C1F	0.707	0.618	0.645	0.653	[2]
**27**	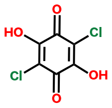	O=C1C(O)=C(Cl)C(=O)C(=C1Cl)O	0.394	0.371	0.374	0.381	[2]

**28**	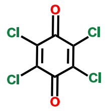	O=C1C(Cl)=C(Cl)C(=O)C(Cl)=C1Cl	0.700	0.641	0.653	0.659	[2]
**29**	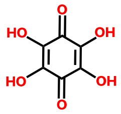	O=C1C(O)=C(O)C(=O)C(O)=C1O	0.280	0.368	0.334	0.334	[2]
**30**	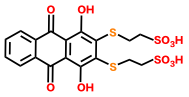	O=S(=O)(O)CCSc(c1O)c(SCCS(=O)(=O)O)c(O)c(c12)C(=O)c3c(C2=O)cccc3	0.113	0.185	0.085	0.102	[3]
**31**	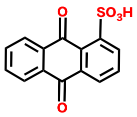	c1ccc(S(=O)(=O)O)c(c12)C(=O)c3c(C2=O)cccc3	0.195	0.257	0.182	0.183	[3]
**32**	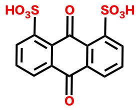	c1ccc(S(=O)(=O)O)c(c12)C(=O)c3c(C2=O)cccc3S(=O)(=O)O	0.206	0.288	0.183	0.196	[3]
**33**	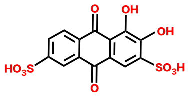	O=S(=O)(O)c(cc1)cc(c12)C(=O)c3c(C2=O)c(O)c(O)c(c3)S(=O)(=O)O	0.133	0.125	0.138	0.130	[3]
**34**	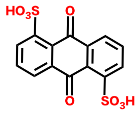	c1ccc(S(=O)(=O)O)c(c12)C(=O)c3c(C2=O)c(S(=O)(=O)O)ccc3	0.239	0.331	0.197	0.199	[3]
**35**	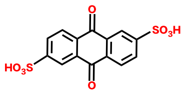	O=S(=O)(O)c(cc1)cc(c12)C(=O)c3c(C2=O)cc(S(=O)(=O)O)cc3	0.228	0.275	0.300	0.360	[3]

**36**	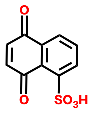	c1ccc(S(=O)(=O)O)c(c12)C(=O)C=CC2=O	0.532	0.509	0.478	0.504	[3]
**37**	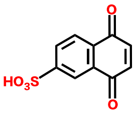	O=S(=O)(O)c(cc1)cc(c12)C(=O)C=CC2=O	0.534	0.498	0.549	0.550	[3]
**38**	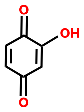	O=C1C=CC(=O)C(O)=C1	0.594	0.487	0.559	0.560	[3]
**39**	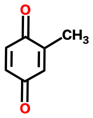	CC1=CC(=O)C=CC1=O	0.641	0.593	0.643	0.640	[3]
**40**	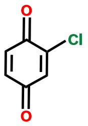	O=C1C=CC(=O)C(Cl)=C1	0.71	0.654	0.678	0.679	[3]
**41**	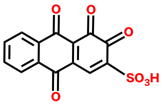	O=c1c(=O)c(S(=O)(=O)O)cc(c2=O)c1c(=O)c(c23)cccc3	1.21	1.098	1.119	1.072	[3]
**42**	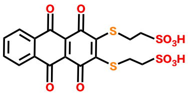	c1cccc(c12)c(=O)c3c(c2=O)c(=O)c(SCCS(=O)(=O)O)c(c3=O)SCCS(=O)(=O)O	1.08	1.100	1.106	1.108	[3]
**43**	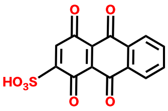	O=c1c(S(=O)(=O)O)cc(=O)c(c2=O)c1c(=O)c(c23)cccc3	1.05	1.239	1.222	1.228	[3]

Table S2. Performance comparisons of 11 different exchange-correlation functionals for the prediction of experimentally measured redox potentials. DFT_g represents both OPT and SPE in the gas-phase; DFT_s represents OPT in the gas-phase followed by SPE in SOL; DFT_{aq} represents both OPT and SPE in SOL.

Scheme for OPT and SPE	DFT_g		DFT_s		DFT_{aq}	
DFT methods	R^2	RMSE (V)	R^2	RMSE (V)	R^2	RMSE (V)
LDA	0.926	0.091	0.935	0.085	-	-
PBE	0.954	0.072	0.977	0.051	0.975	0.053
PBE-D3	0.953	0.073	0.977	0.050	-	-
BLYP	0.960	0.067	0.979	0.048	-	-
BLYP-D3	0.958	0.068	0.980	0.047	-	-
B3LYP	0.966	0.062	0.982	0.045	0.979	0.049
B3LYP-D3	0.964	0.064	0.982	0.045	-	-
PBE0	0.966	0.062	0.983	0.043	-	-
PBE0-D3	0.965	0.063	0.984	0.043	-	-
HSE06	0.967	0.061	0.983	0.044	-	-
M08-HX	0.971	0.057	0.983	0.044	0.981	0.047

Table S3. Performance comparisons of nine different SEQM methods for the prediction of experimentally measured redox potentials. $SEQM_g$ represents OPT or SPE in gas-phase. PBE_g, $B3LYP_g$ and $M08\text{-}HX_g$, represent SPE calculations in gas-phase using these three exchange-correlation functionals.

Scheme for OPT and SPE	OPT:$SEQM_g$ SPE:$SEQM_g$		OPT:$SEQM_g$ SPE:PBE_g		OPT:$SEQM_g$ SPE:$B3LYP_g$		OPT:$SEQM_g$ SPE:$M08\text{-}HX_g$	
Method	R^2	RMSE(V)	R^2	RMSE(V)	R^2	RMSE(V)	R^2	RMSE(V)
AM1	0.899	0.107	0.963	0.064	0.958	0.068	0.955	0.071
MNDO	0.773	0.159	0.840	0.134	0.852	0.129	0.860	0.125
MNDOD	0.748	0.168	0.847	0.131	0.860	0.125	0.867	0.122
PM3	0.864	0.123	0.952	0.073	0.957	0.070	0.956	0.070
PM6	0.863	0.124	0.928	0.090	0.916	0.097	0.891	0.111
PM6-D3	0.870	0.121	0.919	0.095	0.907	0.102	0.895	0.108
PM6-D3H4X	0.877	0.117	0.947	0.077	0.943	0.080	0.933	0.087
PM7	0.906	0.103	0.954	0.072	0.961	0.066	0.967	0.061
RM1	0.846	0.131	0.958	0.069	0.956	0.071	0.952	0.074

Table S4. Performance comparisons of nine different SEQM methods for the prediction of experimentally measured redox potentials. $SEQM_g$ represents OPT or SPE in gas-phase. PBE_s, $B3LYP_s$ and $M08\text{-}HX_s$, represent SPE calculations in SOL using these three exchange-correlation functionals.

Scheme for OPT and SPE	OPT:$SEQM_g$ SPE:$SEQM_g$		OPT: $SEQM_g$ SPE: PBE_s		OPT: $SEQM_g$ SPE: $B3LYP_s$		OPT: $SEQM_g$ SPE: $M08\text{-}HX_s$	
Method	R^2	RMSE(V)	R^2	RMSE(V)	R^2	RMSE(V)	R^2	RMSE(V)
AM1	0.899	0.107	0.969	0.059	0.967	0.061	0.960	0.067
MNDO	0.773	0.159	0.892	0.110	0.902	0.105	0.906	0.102
MNDOD	0.748	0.168	0.888	0.112	0.898	0.107	0.902	0.105
PM3	0.864	0.123	0.975	0.053	0.976	0.052	0.974	0.054
PM6	0.863	0.124	0.916	0.097	0.892	0.110	0.849	0.130
PM6-D3	0.870	0.121	0.920	0.095	0.880	0.116	0.867	0.122
PM6-D3H4X	0.877	0.117	0.949	0.076	0.931	0.088	0.905	0.103
PM7	0.906	0.103	0.976	0.051	0.972	0.056	0.969	0.059
RM1	0.846	0.131	0.959	0.068	0.953	0.072	0.940	0.082

Table S5. Performance comparisons of nine different SEQM methods for the prediction of experimentally measured redox potentials. $SEQM_{aq}$ represents OPT or SPE in SOL. PBE_g, $B3LYP_g$ and $M08\text{-}HX_g$, represent SPE calculations in gas-phase with three exchange-correlation functionals.

Scheme for OPT and SPE	OPT:$SEQM_{aq}$ SPE:$SEQM_{aq}$		OPT: $SEQM_{aq}$ SPE: PBE_g		OPT: $SEQM_{aq}$ SPE: $B3LYP_g$		OPT: $SEQM_{aq}$ SPE: $M08\text{-}HX_g$	
Method	R^2	RMSE(V)	R^2	RMSE(V)	R^2	RMSE(V)	R^2	RMSE(V)
AM1	0.886	0.113	0.956	0.070	0.947	0.077	0.937	0.084
MNDO	0.394	0.261	0.654	0.197	0.649	0.198	0.563	0.221
MNDOD	0.820	0.142	0.772	0.160	0.793	0.152	0.812	0.145
PM3	0.861	0.125	0.925	0.092	0.930	0.089	0.930	0.088
PM6	0.927	0.090	0.919	0.095	0.919	0.095	0.914	0.098
PM6-D3	0.926	0.091	0.934	0.086	0.925	0.092	0.919	0.095
PM6-D3H4X	0.909	0.101	0.929	0.089	0.923	0.093	0.918	0.096
PM7	0.915	0.098	0.943	0.080	0.944	0.079	0.950	0.075
RM1	0.869	0.121	0.928	0.090	0.928	0.090	0.923	0.093

Table S6. Performance comparisons of nine different SEQM methods for the prediction of experimentally measured redox potentials. $SEQM_{aq}$ represents OPT or SPE in SOL. PBE_s, $B3LYP_s$ and $M08\text{-}HX_s$, represent SPE calculations in SOL with three exchange-correlation functionals.

Scheme for OPT and SPE	OPT:$SEQM_{aq}$ SPE:$SEQM_{aq}$		OPT: $SEQM_{aq}$ SPE: PBE_s		OPT: $SEQM_{aq}$ SPE: $B3LYP_s$		OPT: $SEQM_{aq}$ SPE: $M08\text{-}HX_s$	
Method	R^2	RMSE (V)	R^2	RMSE(V)	R^2	RMSE(V)	R^2	RMSE(V)
AM1	0.886	0.113	0.961	0.066	0.951	0.074	0.941	0.082
MNDO	0.394	0.261	0.815	0.144	0.796	0.151	0.846	0.131
MNDOD	0.820	0.142	0.870	0.121	0.886	0.113	0.901	0.105
PM3	0.861	0.125	0.957	0.069	0.963	0.065	0.965	0.062
PM6	0.927	0.090	0.940	0.082	0.938	0.084	0.926	0.091
PM6-D3	0.926	0.091	0.953	0.073	0.940	0.082	0.927	0.090
PM6-D3H4X	0.909	0.101	0.948	0.076	0.941	0.081	0.937	0.084
PM7	0.915	0.098	0.962	0.065	0.961	0.066	0.967	0.060
RM1	0.869	0.121	0.947	0.077	0.944	0.079	0.928	0.090

Table S7. Performance comparisons of FF(OPLS3e) in combination with different DFT methods for the prediction of experimentally measured redox potentials. FF_g represents OPT or SPE in gas-phase. FF_{aq} represents OPT or SPE in SOL. PBE_g, $B3LYP_g$ and M08-HX_g, represent SPE calculations in gas-phase with three DFT functionals. PBE_s, $B3LYP_s$ and M08-HX_s represent SPE calculation in SOL with three exchange-correlation functionals.

Scheme for OPT and SPE	OPT:FF_g SPE:FF_g		OPT: FF_g SPE: PBE_g		OPT: FF_g SPE: $B3LYP_g$		OPT: FF_g SPE: M08-HX_g	
Method	R^2	RMSE(V)	R^2	RMSE(V)	R^2	RMSE(V)	R^2	RMSE(V)
OPLS3e	0.596	0.213	0.947	0.077	0.945	0.079	0.936	0.085
Scheme for OPT and SPE	OPT:FF_g SPE:FF_g		OPT: FF_g SPE: PBE_s		OPT: FF_g SPE: $B3LYP_s$		OPT: FF_g SPE: M08-HX_s	
Method	R^2	RMSE(V)	R^2	RMSE(V)	R^2	RMSE(V)	R^2	RMSE(V)
OPLS3e	0.596	0.213	0.969	0.059	0.957	0.070	0.945	0.078
Scheme for OPT and SPE	OPT:FF_{aq} SPE:FF_{aq}		OPT: FF_{aq} SPE: PBE_g		OPT: FF_{aq} SPE: $B3LYP_g$		OPT: FF_{aq} SPE: M08-HX_g	
Method	R^2	RMSE(V)	R^2	RMSE(V)	R^2	RMSE(V)	R^2	RMSE(V)
OPLS3e	0.06	0.325	0.939	0.083	0.933	0.087	0.926	0.091
Scheme for OPT and SPE	OPT:FF_{aq} SPE:FF_{aq}		OPT: FF_{aq} SPE: PBE_s		OPT: FF_{aq} SPE: $B3LYP_s$		OPT: FF_{aq} SPE: M08-HX_s	
Method	R^2	RMSE(V)	R^2	RMSE(V)	R^2	RMSE(V)	R^2	RMSE(V)
OPLS3e	0.06	0.325	0.964	0.063	0.951	0.074	0.938	0.083

Table S8. Performance comparisons of two different DFTB methods for the prediction of experimentally measured redox potentials. $DFTB_g$ represents OPT or SPE in gas-phase. PBE_g, $B3LYP_g$ and $M08\text{-}HX_g$, represent SPE calculations in gas-phase with three exchange-correlation functionals.

Scheme for OPT and SPE	OPT:$DFTB_g$ SPE:$DFTB_g$		OPT: $DFTB_g$ SPE: PBE_g		OPT: $DFTB_g$ SPE: $B3LYP_g$		OPT: $DFTB_g$ SPE: $M08\text{-}HX_g$	
Method	R^2	RMSE(V)	R^2	RMSE(V)	R^2	RMSE(V)	R^2	RMSE(V)
DFTB-D3	0.953	0.072	0.960	0.067	0.964	0.063	0.964	0.063
GFN1-xTB	0.944	0.079	0.949	0.075	0.961	0.066	0.965	0.062

Table S9. Performance comparisons of two different DFTB methods for the prediction of experimentally measured redox potentials. $DFTB_g$ represents OPT or SPE in gas-phase. PBE_s, $B3LYP_s$ and $M08\text{-}HX_s$, represent SPE calculations in SOL with three exchange-correlation functionals.

Scheme for OPT and SPE	OPT:$DFTB_g$ SPE:$DFTB_g$		OPT: $DFTB_g$ SPE: PBE_s		OPT: $DFTB_g$ SPE: $B3LYP_s$		OPT: $DFTB_g$ SPE: $M08\text{-}HX_s$	
Method	R^2	RMSE(V)	R^2	RMSE(V)	R^2	RMSE(V)	R^2	RMSE(V)
DFTB-D3	0.953	0.072	0.978	0.049	0.974	0.054	0.968	0.060
GFN1-xTB	0.944	0.079	0.977	0.051	0.981	0.046	0.976	0.052

References for Supporting Information

1. Gerhardt, M. R. et al. Anthraquinone Derivatives in Aqueous Flow Batteries. *Adv. Energy Mater.* **7**, 1601488 (2017).
2. Wedege, K., Dražević, E., Konya, D. & Bentien, A. Organic Redox Species in Aqueous Flow Batteries: Redox Potentials, Chemical Stability and Solubility. *Sci. Rep.* **6**, 1–13 (2016).
3. Tabor, D. P. *et al.* Mapping the frontiers of quinone stability in aqueous media: implications for organic aqueous redox flow batteries. *J. Mater. Chem. A* **7**, 12833–12841 (2019).
4. Tobias Johnsson Wass, J. R., Ahlberg, E., Panas, I. & Schiffrin, D. J. Quantum chemical modeling of the reduction of quinones. *J. Phys. Chem. A* **110**, 2005–2020 (2006).
